# Combination Therapy of Polydeoxyribonucleotide and Microcurrent in Muscle Regeneration on Cast-Induced Muscle Atrophy in Rabbit

**DOI:** 10.1155/2022/7469452

**Published:** 2022-10-27

**Authors:** Dong Rak Kwon, Yong Suk Moon, Do Yun Kwon

**Affiliations:** ^1^Department of Rehabilitation Medicine, Muscle Research Center, Catholic University of Daegu School of Medicine, Daegu 42472, Republic of Korea; ^2^Department of Anatomy, Catholic University of Daegu School of Medicine, Daegu 42472, Republic of Korea

## Abstract

**Background:**

The aim of this study was to evaluate how polydeoxyribonucleotide (PDRN) and microcurrent therapy (MT) functioned synergistically in a cast-immobilized rabbit model with an atrophied calf muscle.

**Methods:**

At the age of 12 weeks, 32 male New Zealand rabbits were enrolled in four groups. After 2 weeks of cast-immobilization, 4 procedures were performed on atrophied calf muscle [weekly two injections normal saline 0.2 ml injection group 1 (G1-NS), weekly two injections 0.2 ml PDRN injection group 2 (G2-PDRN), MT group 3 (G3-MT), and 0.2 ml PDRN injection with MT group 4 (G4-PDRN+MT)]. For 2 weeks, MT was used for 60 minutes each day. The calf circumference (CC), the thickness of gastrocnemius muscle (TGCM), and the tibial nerve compound muscle action potential (CMAP) were evaluated using ultrasound before and after 2 weeks of treatment. Proliferating cell nuclear antigen (PCNA), vascular endothelial growth factor, and platelet endothelial cell adhesion molecule-1 (PECAM-1) of GCM fibers (type I, type II, and total) were measured. Statistical analyses were performed using ANOVA.

**Results:**

The mean atrophic alterations of right CC, CMAP, and TGCM (medial/lateral) were substantially lower in G4-PDRN+MT than in the G1-NS, G2-PDRN, and G3-MT, respectively (*p* < 0.05). Furthermore, mean CSAs (type I, type II, and total) of medial and lateral GCM muscle fibers in G4-PDRN+MT were significantly higher when compared to other three groups (*p* < 0.05). In terms of the PCNA-, VEGF-, and PECAM-1-positive cell ratio of medial and lateral GCM muscle fibers, G4-PDRN+MT was considerably higher than G1-NS, G2-PDRN, and G3-MT (*p* < 0.05).

**Conclusions:**

On the atrophied calf muscle of the rabbit model, PDRN injection combined with MT was more effective than PDRN injection alone, MT alone, and normal saline injection separately.

## 1. Introduction

Skeletal muscle dysfunction/atrophy, regardless of the disease, restricts a patient's quality of life and everyday activities [1]. The most relevant factors leading to a patient's muscular atrophy and function loss are deconditioning and decreased muscle activities [1, 2]. Immobilization, in particular, can cause skeletal muscle atrophy and deterioration when used to treat soft-tissue and bone injuries [3, 4]. It also caused significant muscle remodeling, including vascular and neural changes, myofibrillar protein changes, and metabolic enzyme activity changes [5]. During an immobilization processes, a short drop in protein synthesis facilitates the rapid loss of myofibrillar protein, which is followed by an increase in protein degradation, which eventually leads to the loss of net protein [6]. This means that the effectiveness of skeletal muscle recovery relies on protein synthesis, irrespective of the cause of muscle atrophy.

Furthermore, satellite cells (i.e., myogenic precursor cells) are also triggered and undergo cellular division in response to muscle injury [7], and myogenic precursor cells operate to repair/regenerate adult skeletal muscle [8–10]. Nonetheless, myogenic precursor cells proliferate in injured skeletal muscle, which may be aided or hampered by extracellular stimuli, e.g., mechanical stress and immobility [11, 12]. Vascular endothelial growth factor (VEGF) has a crucial role in the construction and function of locomotive skeletal muscle, and its variations have an impact on the overall functioning of the organism, including motor tolerance [13, 14]. In addition, during undertaking exercise training, skeletal muscle remodeling largely depends on the appropriate expression of VEGF in various tissues such as skeletal myofibers [15, 16].

A recent study [17] found that merely 2 weeks of leg immobilization causes a significant reduction in angiogenesis capacity, as indicated by lower muscle and platelet VEGF concentration. The researchers believed that specific therapies (e.g., passive movement of the wounded limb or training with the non-injured limb) can help sustain angiogenesis potential while the injured limb remains immobile. It is consistent with lower VEGF levels in the skeletal muscle of elderly patients. [18]. Because VEGF stored in myocytes is essential to skeletal muscle angiogenesis [19], this loss of VEGF appears to be critical.

Polydeoxyribonucleotide (PDRN), a DNA-derived medication that contained a combination of deoxyribonucleotide polymers with chain lengths between 50 and 2000 bp, has recently been found to be effective in the management of chronic rotator cuff disorders [20]. Angiogenesis and collagen synthesis are two of PDRN's main characteristics, and it also exerts anti-inflammatory activity [21]. Concomitant injection of umbilical cord blood-derived mesenchymal stem cells (UCB-MSCs) and PDRN was more efficient than UCB-MSC injection alone in enhancing full thickness RCTT (FTRCTT) in a recent animal study [22]. The impact is thought to be mediated via increasing VEGF, which has restorative potential in RCTT. As a major growth factor, VEGF's stimulation of the formation of new blood vessels in areas where poor circulation persists, such as FTRCTT, accelerates the healing process. In fact, via regulating many biological processes of endothelial cells, VEGF enhances the synthesis of vasodilating mediators, increases vascular permeability, and stimulates migration, proliferation, and formation [23].

Microcurrent theory (MT) is used to explain how a therapeutic application of a low-frequency current (i.e., <l mA) creates sensory level stimulation. MT has already been shown to induce muscle cell regeneration and slow the onset of GCM muscle atrophy [24, 25]. This suggests that MT can stimulate satellite cell proliferation while also increasing protein synthesis. That is, MT should be pursued as an effective therapeutic approach for muscle atrophy caused by protracted immobility.

Microcurrent stimulation has also been proven to enhance the release of VEGF in vitro animal model and in clinical practice [26, 27]. MT increases the generation of adenosine triphosphate (ATP), which is a necessary energy source for a wide range of intracellular metabolic processes. It can assist in providing the high energy supply [28, 29] essential for the complex wound healing process, allowing the process to be hastened. With this background, we might hypothesize that local injections of PDRN and MT, rather than PDRN administration in animal models, may be more effective in healing muscle atrophy. To test this theory, we used rabbits immobilized-by-cast (IC) to evaluate the effect of MT-bound PDRN on gastrocnemius (GCM) muscle atrophy regeneration.

## 2. Methods

### 2.1. Animal Grouping

Our study protocol was approved by the Institutional Animal Care and Use Committee (IACUC) of the Catholic University of Daegu School of Medicine (IRB no.: DCIAFCR-200921-09-Y), in accordance with IACUC guidelines for animal care/use. We enrolled male New Zealand White rabbits (*n* = 32, 12 weeks old), with a mean of 3.3 kg (2.8-3.6 kg) in separate steel cages and at constant temperature/humidity (23 ± 2°C and 45 ± 10%, respectively). Free access to tap water was allowed for all rabbits which were also fed a (commercial) rabbit diet [24, 25].

**Figure 1:**
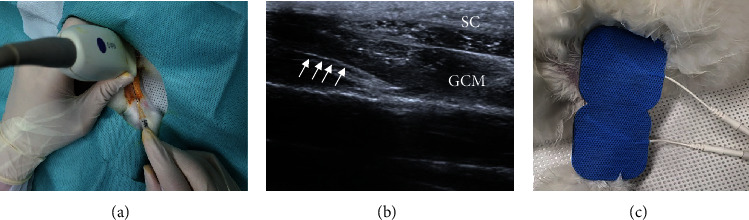
Microcurrent application and PDRN injection under ultrasound guidance. The injection of 0.2 ml PDRN (a) into the gastrocnemius muscle (white arrows indicating injection needle) under ultrasound guidance (b) and MT was performed (c). MT: microcurrent therapy; PDRN: polydeoxyribonucleotide; SC: subcutaneous tissue; GCM: medial gastrocnemius muscle.

They were allocated into 4 groups (*n* = 8 per group) using computerized random numbers after 2 weeks of IC. The following 4 types of procedures were performed at atrophied calf muscle: group 1 with weekly two injections normal saline 0.2 ml injection (G1-NS), group 2 with weekly two injections 0.2 ml PDRN injection (G2-PDRN), group 3 with microcurrent therapy (G3-MT), and group 4 with weekly two injections 0.2 ml PDRN injection with microcurrent therapy (G4-PDRN+MT) (Figure 1). We followed a physiatrist's ultrasound (US) standards for all injections, and the sonographer had an 18-year experience in musculoskeletal US. A commercially accessible US system with a 5-18 MHz multifrequency linear transducer (EPIQ 5; Philips Healthcare, Andover, MA, USA) was used. We did not give the rabbits any medication, and they were all euthanized two weeks following the first injection.

### 2.2. Immobilized-By-Cast (IC)

For 2 weeks, we applied IC on the right GCM muscles. We used a PVC plastic splint, a nonadhesive bandage, and an adhesive elastic bandage (Tensoplast®; Smith & Nephew Medical, London, UK) to extend the rabbits' right knees and ankles as per the standardized IC technique. The procedures of Kauhanen et al. [30] were used in this study. Muscle samples were obtained from the right hind limb GCM muscle two weeks after therapy for microscopic evaluation using the techniques developed by Kwon, et al. [24] and Park et al. [25]. The contralateral side is loaded due to the unilateral immobility of the hind limb. The left limbs were not used as controls. All procedures were conducted under general anesthesia using intramuscular Zoletil® 50 (15 mg/kg, Virbac Korea, Seoul, Korea) and xylazine (5 mg/kg, Rompun®; Bayer Co., Seoul, Korea) injections. We also removed the hind limb hair of all the rabbits using a hair remover [25].

### 2.3. Injection Procedures

All injections were done under anesthesia with Zoletil® 50 (15 mg/kg, Virbac Korea, Seoul, Korea) and xylazine (5 mg/kg, Rompun®; Bayer Co., Seoul, Korea) via intramuscular injection. We applied ultrasound-guided normal saline/PDRN injections (0.1 ml, respectively) at two points, i.e., lateral and medial sides of the gastrocnemius (GCM) muscle, using a 5–13-MHz linear transducer (Antares; Siemens Healthcare, Erlangen, Germany) (Figures 1(a) and 1(b)). Commercially obtained PDRN (Rejuvenex Inj., polydeoxyribonucleotide sodium, 5.625 mg/3 ml, Pharma Research Product, South Korea) was used. 0.1 ml from each solution was injected into the lateral and medial sides on the same (horizontal) line that was based on a middle reference point (a total of 0.2 ml at two points). The midpoint between the following two points was determined as the middle reference point: proximal one-third point of the longitudinal line between the midpoint of ankle malleoli and the midpoint of femoral epicondyles and the medial and lateral endpoints of a transverse line drawn perpendicularly to the point of the longitudinal line. Same-site repeat injections were done one week later.

### 2.4. Microcurrent Therapy (MT)

The MT generator (alternating current, 25 *μ*A, 8 Hz) was configured to supply “a single square pulse formatted current” that reversed polarity every 3 seconds. We apply an MT electric patch to each rabbit's skin, passing it through the GCM muscle proximally (anode) and distally (cathode). Under anesthesia (i.e., ketamine/xylazine), we used microcurrent to activate the GCM muscles of G3-MT and G4-PDRN+MT for 60 minutes every day for 2 weeks (Figure 1). We did not observe any muscle contractions in the rabbit's hind limbs during the MT period. The rabbits were otherwise free to roam in the cage. Prior to euthanasia, the size of the calf, the complex muscle action potential (CMAP) of the tibial nerve, and the thickness of the middle GCM muscle were all measured by ultrasound. We used the methods developed by Moon et al. [24] and Park et al. [25].

### 2.5. Clinical Parameters

The same physiatrist, blinded to the groups and with a 23-year experience in electrophysiology, measured all the clinical parameters. The largest calf circumference was measured using a tape while the knees were flexed at 90° and the ankles were relaxed. We followed the methods of Moon et al. [24] and Park et al. [25].

To avoid anisotropic aberrations during the ultrasonic measurements, the ultrasound probe was kept parallel to the muscle fibers [24, 25]. We evaluated the median GCM muscle thickness and calf circumference when the rabbits' knees were bent 90 degrees to relax the ankles. In the midpoint of the two reference positions, we took longitudinal ultrasound images at a fixed point on the midupper side of the GCM muscle. The first reference point was set on the proximal 1/3 of the vertical line from the midpoint between the malleolus on both sides of the ankle to the midpoint between the epicondyle of both femurs, whereas the second reference point was located on the middle end of the horizontal line perpendicular to the point of the longitudinal line [24, 25]. We used real-time ultrasonography to assess the medial GCM muscle thickness between the surface and the deep fascia and a tape to determine the largest calf circumference.

The active recording electrode was positioned on the GCM muscles' midway surface, while the reference electrode was put on the ankle's subcutaneous tissue [24, 25]. We electrically stimulated the tibial nerve at the popliteal fossa, recorded the greatest ultimate CMAPs after 8-10 supramaximal shocks, and used the following equation: Lt.side − Rt.side/Lt.side × 100, to calculate atrophic changes in right calf circumference, tibial nerve CMAP, and GCM muscle fiber thickness [24, 25].

### 2.6. Tissue Preparation

An anatomist, blinded to the groups, measured all the study's histological parameters. All the rabbits were euthanized, as previously stated. We used neutral buffered formalin to segment and fix all the rabbits' medial and later GCM muscle fibers for 48 hours, then embedded the specimens in paraffin (Paraffin; Oxford, St. Louis, MO, USA), and cut it horizontally into serial sections, each 5 *μ*m thick. We followed the methods developed by Kwon, et al. [24] and Park et al. [25].

### 2.7. Immunohistochemistry

We used monoclonal anti-myosin antibodies (Skeletal, Slow; Sigma-Aldrich, St. Louis, MO, USA) and monoclonal anti-myosin antibodies (skeletal, Fast; Sigma-Aldrich, St. Louis, MO, USA) to stain the muscle sections for type I fibers and type II fibers, respectively. In addition, we used monoclonal proliferation-preventing cell nuclear antigen monoclonal antibody (PCNA, PC10; Santa Cruz Technologies, Dallas, TX, USA) to immunostain the sections of the markers of satellite cells proliferating, followed by anti-vascular endothelial growth factor polyclonal antibody (VEGF, A-20; Santa Cruz Biotechnology) and anti-platelet endothelial cell adhesion molecule-1 polyclonal antibody (PECAM-1, M-20; Santa Cruz Biotechnology). The slides were then washed in phosphate-buffered saline (PBS) for immunohistochemistry [22, 25].

Endogenous peroxidases were inactivated for 30 minutes in PBS containing 0.3% H_2_O_2_, and nonspecific protein binding was blocked for 30 minutes in PBS containing 10% normal horse serum (Vector Laboratories, Burlingame, CA, USA). The sections were incubated in primary antibodies (1 : 100~1 : 500) for 2 h at room temperature before being washed 3 times with PBS. The secondary antibody was applied to the muscle sections for 1 hour at room temperature prior being washed 3 times with PBS. The avidin-biotin-peroxidase complex (ABC; Vector Laboratories) was applied on the sections for 1 hour and rinsed 3 times with PBS, before performing a peroxidase reaction with 0.05 M Tris-HCl (pH 7.6) containing 0.01% H_2_O_2_ and 0.05% 3,3′-diaminobenzidine (DAB; Sigma-Aldrich). Finally, we used an Axiophot Photomicroscope (Carl Zeiss, Oberkochen, Germany) and AxioCam MRc5 (Carl Zeiss) to mount and analyze the slides [22, 25].

### 2.8. Histomorphometric Analysis

Type I muscle fibers are more impacted by GCM atrophy caused by immobility than type II muscle fibers, according to a previous study [24, 25]; hence, we focused our analyses on type I muscle fibers in this study. We stressed the type 1 and type II as previously described. Our study's histologic analysis was performed by an anatomist with 20 years of professional experience in histologic examination and who was unaware of the group allocation. We used a Carl Zeiss Axiophot Photomicroscope to analyze the slides and an AxioCam MRc5 (Carl Zeiss) to acquire images of 5 randomly chosen areas from the groups. We (a) used the antimyosin immunostained muscle sections from the digital images (x100) to identify the entire muscle cross section; (b) used the image morphometry program (AxioVision SE64; Carl Zeiss) to trace antimyosin positive type I or II muscle fiber cross-sectional area (CSA); and (c) measured the average CSA of type I or type II muscle fibers [24, 25].

### 2.9. Evaluation of Immunohistochemical Staining

We used an AxioCam MRc5 (Carl Zeiss) interfaced with an Axiophot Photomicroscope (Carl Zeiss) to examine anti-PCNA, VEGF, and PECAM-1 immunostaining slides. We randomly photographed twenty chosen areas from the groups and analyzed them with the AxioVision SE64 (Carl Zeiss) program [24, 25]. On each image, we counted the total number of anti-PCNA-, VEGF-, and PECAM-1-positive cells or nuclei as well as the total number of muscle fibers. The PCNA, VEGF, and PECAM-1 ratio was then calculated by multiplying the number of anti-PCNA-, VEGF-, and PECAM-1-positive cells or nuclei by 1,000 muscle fibers.

### 2.10. Statistical Analysis

Statistical analyses were done using SPSS, version 25.0 (SPSS Inc., Chicago, IL, USA). A pilot study was conducted for calculating the sample size. The primary end point was the total muscle fiber CSA of medial GCM. In the pilot study, each group comprised one rabbit; thus, three randomly chosen areas were evaluated in each group. The effect size was 0.49; accordingly, a minimum of 76 fields were required to achieve a power of at least 95% with 0.05 significance. Three fields could be taken from one rabbit; therefore, 26 rabbits were necessary. Considering a rate of 20%, a sample size of 32 was determined. Aside from descriptive statistics (i.e., mean and standard deviation), we employed ANOVA to detect differences within/between groups. Furthermore, we utilized Tukey's test if the ANOVA analysis indicated significant differences between groups. The mean was followed by the 95% confidence interval, and all data were expressed as the mean ± standard deviation (SD). Statistical significance was set at *p* < 0.05, and the power was estimated to exceed 0.95 through post hoc power analysis.

## 3. Results

We found significant differences in clinical, imaging, and electrophysiologic parameters across the four groups (*p* < 0.05, Table 1). For the four dimensions including right calf circumference, amplitude of CMAP of the right tibial nerve, right medial GCM, and lateral GCM muscle thickness, we revealed that G2-PDRN (19.3 ± 1.4, 21.9 ± 1.7, 14.8 ± 0.9, and 14.6 ± 0.7) and G3-MT (19.9 ± 1.5, 21.5 ± 1.8, 15.1 ± 0.1, and 14.8 ± 0.6) were significantly lower than G1-NS: (23.0 ± 1.6, 23.2 ± 1.7, 16.3 ± 0.8, and 15.9 ± 0.6), respectively (*p* < 0.05, Table 1). Interestingly, G4-PDRN+MT had considerably less atrophic alterations in the right calf circumference including CMAP of tibial nerve, thickness of medial, and lateral GCM muscle than those in the other three groups (*p* < 0.05, Table 1). Furthermore, no significant differences were in clinical parameters between G2-PDRN and G3-MT.

**Figure 2:**
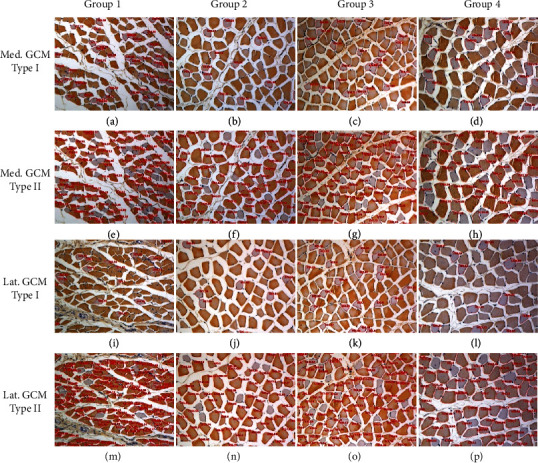
Muscle sections were immunohistochemically stained for muscle fiber. The cross-sectional area (red circle) of gastrocnemius type 1 (a–d) and type 2 (e–h) muscle fiber was measured by image morphometry. Muscle fiber atrophy was seen in group 1 (a, e). Muscle fiber cross-sectional area was increased in group 2 (b, f) group 3 (c, g), and group 4 (d, h) as compared to group 1. Group 1: IC for 2 weeks and NS injection for 2 weeks after CR; group 2: IC for 2 weeks and PDRN injection for 2 weeks after CR; group 3: IC for 2 weeks and MT for 2 weeks after CR; group 4: IC for 2 weeks and PDRN injection and MT for 2 weeks after CR; IC: immobilized by cast; CR: cast removal; NS: normal saline; PDRN: polydeoxyribonucleotide; MT: microcurrent therapy.

There was a significant difference in histological parameters between the 4 groups (*p* < 0.05, Figure 2, Table 2). G2-PDRN (633.59 ± 123.2 *μ*m^2^, 955.08 ± 350.1 *μ*m^2^, and 888.29 ± 342.4 *μ*m^2^), G3-MT (505.21 ± 155.4 *μ*m^2^, 817.67 ± 231.2 *μ*m^2^, and 752.99 ± 251.8 *μ*m^2^), and G4-PDRN+MT (863.73 ± 225.0 *μ*m^2^, 1,306.06 ± 393.5 *μ*m^2^, and 1,192.61 ± 406.6 *μ*m^2^) all had significantly higher mean CSAs of type I, type II, and total medial GCM muscle fibers than G1-NS (287.63 ± 92.6 *μ*m^2^, 443.09 ± 121.2 *μ*m^2^, and 399.03 ± 133.7 *μ*m^2^) (*p* < 0.05, Table 2). Furthermore, we found that in G2-PDRN and G3-MT, the mean CSAs of type I, type II, and total medial and lateral GCM muscle fibers were significantly higher than those in G1-NS (*p* < 0.05, Table 2). In addition, we observed that the mean CSAs of type I medial and lateral GCM muscle fibers in G4-PDRN+MT were the largest of the four groups (*p* < 0.05, Table 2) with no significant differences in immunohistochemical findings between G2-PDRN and G3-MT.

**Figure 3:**
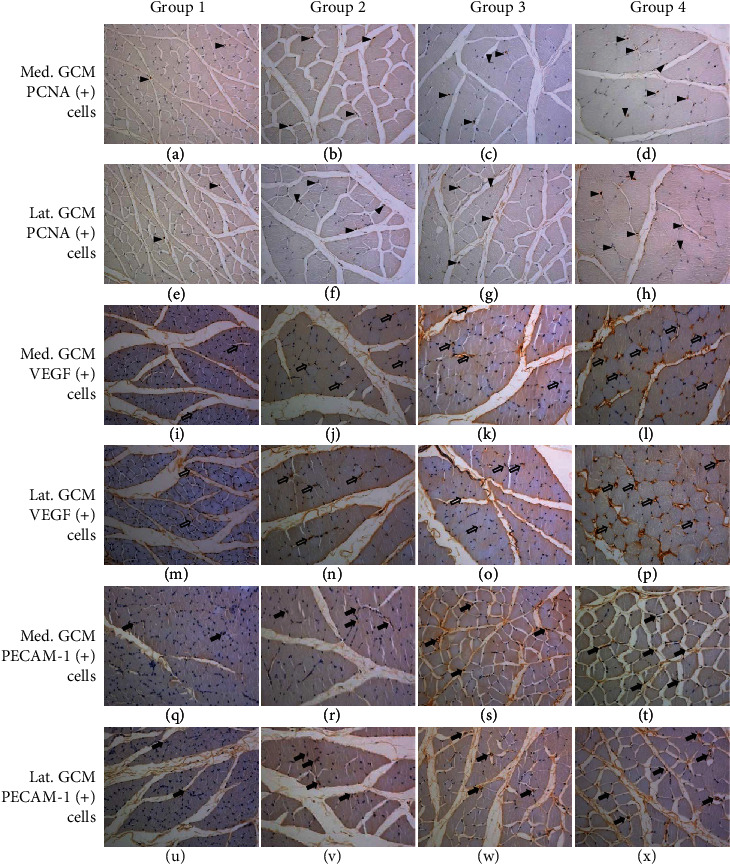
Immunohistochemical findings in groups 1-4. PCNA-positive cells were observed in the medial/lateral gastrocnemius muscle fibers (arrowheads). PCNA-positive cells or nuclei were significantly abundant in group 4 when compared to groups 1-3. VEGF-positive cells were observed in the medial/lateral gastrocnemius muscle fibers (open arrows). VEGF-positive cells or nuclei were significantly abundant in group 4 when compared to groups 1-3. PECAM-1-positive cells were found in the medial/lateral gastrocnemius muscle fibers (closed arrows). PECAM-1-positive cells or nuclei were significantly abundant in group 4 vs. groups 1-3. Group 1: IC for 2 weeks and NS injection for 2 weeks after CR; group 2: IC for 2 weeks and PDRN injection for 2 weeks after CR; group 3: IC for 2 weeks and MT for 2 weeks after CR; group 4: IC for 2 weeks and PDRN injection and MT for 2 weeks after CR; IC: immobilized by cast; CR: cast removal; NS: normal saline; PDRN: polydeoxyribonucleotide; MT: microcurrent therapy.

We were aware that G2-PDRN (0.217 ± 0.068, 0.204 ± 0.039), G3-MT (0.179 ± 0.039, 0.181 ± 0.046), and G4-PDRN+MT (0.386 ± 0.105, 0.374 ± 0.094) were significantly higher than G1-NS (0.076 ± 0.027, 0.063 ± 0.016) for PCNA ratios of medial and lateral GCM muscle fibers (*p* < 0.05, Figure 3, Table 3). In terms of the PCNA ratio of medial and lateral GCM muscle fibers, we noticed that G4-PDRN+MT was significantly higher than G2-PDRN and G3-MT (*p* < 0.05, Table 3). The PCNA ratio of medial and lateral GCM muscle fibers did not differ significantly between G2-PDRN and G3-MT.

We discovered that G2-PDRN (0.282 ± 0.106, 0.301 ± 0.091), G3-MT (0.264 ± 0.108, 0.271 ± 0.062), and G4-PDRN+MT (0.489 ± 0.105, 0.479 ± 0.113) were significantly greater than G1-NS (0.161 ± 0.075, 0.146 ± 0.055) in the case of the VEGF ratio of medial and lateral GCM muscle fibers (*p* < 0.05, Figure 3, Table 3). Furthermore, we discovered that G4-PDRN+MT was significantly higher than G2-PDRN and G3-MT in the case of the VEGF ratio of medial and lateral GCM muscle fibers (*p* < 0.05, Table 3). In addition, between G2-PDRN and G3-MT, there was no significant variation in the VEGF ratio of medial and lateral GCM muscle fibers.

Finally, we found that the PECAM-1 ratios of medial and lateral GCM muscle fibers in G2-PDRN (0.280 ± 0.073, 0.293 ± 0.053), G3-MT (0.265 ± 0.089, 0.279 ± 0.066), and G4-PDRN+MT (0.493 ± 0.075, 0.488 ± 0.133) were significantly higher than those in G1-NS (0.059 ± 0.032, 0.061 ± 0.039) (*p* < 0.05, Figure 3, Table 3). Furthermore, we observed that G4-PDRN+MT was significantly higher than G2-PDRN and G3-MT in the PECAM-1 ratios of medial and lateral GCM muscle fibers (*p* < 0.05, Table 3). Lastly, we noticed no significant difference between G2-PDRN and G3-MT in the PECAM-1 ratio of medial and lateral GCM muscle fibers.

## 4. Discussion

There is a paucity of studies regarding the comparison of the regenerative effects of chemical binding (PDRN) and mechanical (MT) therapy on atrophic skeletal muscle. In this study, we believe that the most important finding is that group 4's (G4-PDRN+MT) atrophic GCM muscles showed a higher regenerative effect than that of the remaining three groups. PDRN combined with MT is more effective than PDRN alone and MT alone in treating atrophy of skeletal muscle caused by IC, according to our findings. This means that, when compared to PDRN alone or MT alone, PDRN with MT, which was supported by clinical, electrophysiological, imaging, and histological parameters, is effective in reducing the negative effects of IC for 2 weeks. This finding also suggests that the adverse effects caused by IC can be minimized for two weeks.

PDRN has been demonstrated to stimulate tissue healing in a variety of medical conditions, including radiodermatitis [31], skin transplant donor sites [32], and tendon regeneration [20, 22]. PDRN has the potential to provide a supply of deoxyribonucleotides and deoxyribonucleosides that can be degraded by active cell membrane enzymes in order to boost cell proliferation and activity in many different tissues [33]. PDRN derivatives can boost nucleic acid synthesis via the salvage pathway [34] and/or by binding to purinergic receptors to respond to various stimuli such as hypoxia [33, 35]. They can also act as growth promoters of fibroblasts [36], pelvis, ECs, and neuroglia [37, 38]. This understanding inspires us to further investigate the effects of PDRN on IC-induced skeletal muscle atrophy.

Furthermore, PDRN stimulates VEGF expression by stimulating the adenosine A_2A_ receptor, and VEGF is an angiogenesis factor that promotes angiogenesis and collagen synthesis [39, 40]. This major growth factor can help hasten the healing process (in degenerative tissue) by promoting the development of new blood vessels in places with inadequate circulation, such as diabetic feet. Specifically, immunohistochemistry staining revealed a larger number of VEGF-positive cells, and the PECAM-1 positive microvascular density of the G4-PDRN+MT group was much greater than that of the other three groups. The fact that MT-induced VEGF expression improved the regenerative effect of PDRN was evidenced by significant differences in clinical, electrophysiology, imaging, and histological parameters in the MT-added and PDRN-alone treated groups. Additional advantages of PDRN include low immunogenicity and safety [22, 41], as well as the availability of pharmaceutical injections. Furthermore, the regeneration action of PDRN is not dose-dependent [41], which indicates that increasing its volume does not increase its therapeutic benefit, which explains why it must be paired with MT.

In several diseases, such as radiodermatitis [24], skin graft donor sites [20], and photorefractive keratectomy [21], PDRN, a molecule that contains a combination of deoxyribonucleotide polymers of various lengths, has been proven to be a tissue repair-stimulating agent. Active cell membrane enzymes are expected to cleave PDRN, producing a supply of deoxyribonucleotides and deoxyribonucleosides that can boost cell proliferation and activity in several tissues [26]. Overall, PDRN derivatives may boost nucleic acid synthesis via salvage pathways [28] and/or binding to the purinergic receptors in response to various stimuli, like hypoxia [22, 26]. They could also operate as growth promoters for fibroblasts [17], osteoblasts, ECs, and neuroglia [18, 27]. All of these factors guided us to investigate the role of PDRN in diabetes-impaired wound healing.

MT, like other kinds of electrotherapy, is affected by the intensity. Previous studies have shown that low-intensity electrotherapy can successfully mend damaged tendons and ligaments [42–46]. For example, electric currents (20 to 100 *μ*A) were employed in the collagen matrix in an experimental guinea pig skin lesion, and the maximum fibroblast-growth response was found near the cathode [46]. Another study revealed that a peak current intensity of 50 *μ*A was more efficient than 500 *μ*A in treating chronic tennis elbow symptoms and facilitating tendon normalization [47]. In a similar study on muscle injury, healing mechanisms, such as amino acid transport, triphosphate production, and protein synthesis, were boosted by 30-40% above the control level of an intensity of 100-500 *μ*A; however, these biostimulatory effects were ineffective when the intensity went over 1,000 *μ*A [42]. In fact, treating muscle-related disorders with an intensity of 10–50 *μ*A amplitude has been advised [48]; a low amperage electric current of <500 *μ*A is useful in reducing the severity of muscle symptoms in muscle damage [49–51]; and these are also optimal for ATP generation [42]. These findings support the findings of this study, which show that low-amperage electric currents increase skeletal muscle regeneration more than high-amperage electric currents.

In this study, we confirm that after PDRN with MT, PCNA-positive satellite cells in the atrophied GCM muscle are significantly greater than after PDRN alone and MT alone. In comparison with the previous studies, we used different parameters of MT (i.e., 8 Hz, 25 *μ*A, and 5000 *μ*A), but our findings were consistent with those electrical stimulations with 2–20 Hz and 0.5–20 mA [52] and 0.3 Hz and 10 *μ*A [29] resulting in an effective stimulus to rescue satellite cell loss in disuse muscle atrophy in pigs and rats, respectively.

In this study, we initially detected satellite cell mitotic activity and, at the same time, muscle fiber regeneration following a 2-week immobilization period and remigration of four weeks [30, 50]. Since MT operates at the microamp level and mimics the electrical strength seen in living tissue, we did not find any adverse or untoward event [52, 53].

There were various limitations to our work, as well as implications for future studies. First, we have not applied subject rabbits to exercise; thus, future studies are warranted to assess the concomitant effects of PDRN, MT, and exercise, as well as the impact of (an)aerobic exercises. Second, the study period was short; it is required to evaluate the long-term effects of MT. Finally, the impact of MT needs on getting optimal results at various frequencies and durations must be assessed (e.g., less than 60 minutes or more than several hours).

In conclusion, we confirm that PDRN injection combined with MT is more effective on calf muscle atrophy in a rabbit model than PDRN injection, microcurrent therapy alone, or normal saline injection, as evidenced by improvement of calf circumference, gastrocnemius muscle thickness, CMAP of tibial nerve, CSA of gastrocnemius muscle fibers, PCNA, and VEGF and PECAM-1 ratio.

## Figures and Tables

**Table 1:** Comparison of regenerative effect of clinical parameters among four groups.

Atrophic change (%)				
	Circumference	CMAP	Rt. GCM muscle thickness	
			Medial	Lateral
Group 1	23.0 ± 1.6	23.2 ± 1.7	16.3 ± 0.8	15.9 ± 0.6
Group 2	19.3 ± 1.4^∗^^||^	21.9 ± 1.7^∗^^||^	14.8 ± 0.9^∗^^||^	14.6 ± 0.7^∗^^||^
Group 3	19.9 ± 1.5^†¶^	21.5 ± 1.8^†¶^	15.1 ± 0.1^†¶^	14.8 ± 0.6^†¶^
Group 4	18.3 ± 1.3^‡||¶^	20.0 ± 1.6^‡||¶^	14.3 ± 0.5^‡||¶^	14.1 ± 0.6^‡||¶^

Values are presented as the mean ± standard deviation. Group 1: IC for 2weeks and NS injection for 2 weeks after CR; group 2: IC for 2weeks and PDRN injection for 2 weeks after CR; group 3: IC for 2 weeks and MT for 2 weeks after CR; group 4: IC for 2weeks and PDRN injection and MT for 2 weeks after CR; IC: immobilized by cast; CR: cast removal; NS: normal saline; PDRN: polydeoxyribonucleotide; MT: microcurrent therapy. ^∗^*p* < 0.05 one-way ANOVA, Tukey's post hoc test between groups 1 and 2. ^†^*p* < 0.05 one-way ANOVA, Tukey's post hoc test between groups 1 and 3. ^‡^*p* < 0.05 one-way ANOVA, Tukey's post hoc test between groups 1 and 4. ^||^*p* < 0.05 one-way ANOVA, Tukey's post hoc test between groups 2 and 4. ^¶^*p* < 0.05 one-way ANOVA, Tukey's post hoc test between groups 3 and 4.

**Table 2:** Comparison of immunohistochemical findings in gastrocnemius muscle fiber among four groups.

	Group 1	Group 2	Group 3	Group 4
Medial GCM				
Type I fiber CSA (*μ*m^2^)	287.63 ± 92.6	633.59 ± 123.2^∗^^||^	505.21 ± 155.4^†¶^	863.73 ± 225.0^‡||¶^
Type II fiber CSA (*μ*m^2^)	443.09 ± 121.2	955.08 ± 350.1^∗^^||^	817.67 ± 231.2^†¶^	1,306.06 ± 393.5^‡||¶^
Total muscle fiber CSA (*μ*m^2^)	399.03 ± 133.7	888.29 ± 342.4^∗^^||^	752.99 ± 251.8^†¶^	1,192.61 ± 406.6^‡||¶^
Lateral GCM				
Type I fiber CSA (*μ*m^2^)	286.20 ± 159.7	538.71 ± 162.5^∗^^||^	479.24 ± 107.8^†¶^	844.94 ± 282.8^‡||¶^
Type II fiber CSA (*μ*m^2^)	420.09 ± 155.6	1,063.01 ± 322.2^∗^^||^	784.57 ± 228.7^†¶^	1,210.06 ± 305.5^‡||¶^
Total muscle fiber CSA (*μ*m^2^)	400.73 ± 163.1	973.86 ± 359.8^∗^^||^	744.19 ± 240.0^†¶^	1,140.79 ± 333.5^‡||¶^

Values are presented as the mean ± standard deviation. Group 1: IC for 2weeks and NS injection for 2 weeks after CR; group 2: IC for 2weeks and PDRN injection for 2 weeks after CR; group 3: IC for 2 weeks and MT for 2 weeks after CR; group 4: IC for 2weeks and PDRN injection and MT for 2 weeks after CR; IC: immobilized by cast; CR: cast removal; NS: normal saline; PDRN: polydeoxyribonucleotide; MT: microcurrent therapy. ^∗^*p* < 0.05 one-way ANOVA, Tukey's post hoc test between groups 1 and 2. ^†^*p* < 0.05 one-way ANOVA, Tukey's post hoc test between groups 1 and 3. ^‡^*p* < 0.05 one-way ANOVA, Tukey's post hoc test between groups 1 and 4. ^||^*p* < 0.05 one-way ANOVA, Tukey's post hoc test between groups 2 and 4. ^¶^*p* < 0.05 one-way ANOVA, Tukey's post hoc test between groups 3 and 4.

**Table 3:** Comparison of PCNA, VEGF, and PECAM-1 ratio in medial and lateral GCM among four groups.

	Group 1	Group 2	Group 3	Group 4
Medial GCM				
PCNA ratio	0.076 ± 0.027	0.217 ± 0.068^∗^^||^	0.179 ± 0.039^†¶^	0.386 ± 0.105^‡||¶^
VEGF ratio	0.161 ± 0.075	0.282 ± 0.106^∗^^||^	0.264 ± 0.108^†¶^	0.489 ± 0.105^‡||¶^
PECAM-1 ratio	0.059 ± 0.032	0.280 ± 0.073^∗^^||^	0.265 ± 0.089^†¶^	0.493 ± 0.075^‡||¶^
Lateral GCM				
PCNA ratio	0.063 ± 0.016	0.204 ± 0.039^∗^^||^	0.181 ± 0.046^†¶^	0.374 ± 0.094^‡||¶^
VEGF ratio	0.146 ± 0.055	0.301 ± 0.091^∗^^||^	0.271 ± 0.062^†¶^	0.479 ± 0.113^‡||¶^
PECAM-1 ratio	0.061 ± 0.039	0.293 ± 0.053^∗^^||^	0.279 ± 0.066^†¶^	0.488 ± 0.133^‡||¶^

Values are presented as the mean ± standard deviation. Group 1: IC for 2weeks and NS injection for 2 weeks after CR; group 2: IC for 2weeks and PDRN injection for 2 weeks after CR; group 3: IC for 2 weeks and MT for 2 weeks after CR; group 4: IC for 2weeks and PDRN injection and MT for 2 weeks after CR; IC: immobilized by cast; CR: cast removal; NS: normal saline; PDRN: polydeoxyribonucleotide; MT: microcurrent therapy. ^∗^*p* < 0.05 one-way ANOVA, Tukey's post hoc test between groups 1 and 2. ^†^*p* < 0.05 one-way ANOVA, Tukey's post hoc test between groups 1 and 3. ^‡^*p* < 0.05 one-way ANOVA, Tukey's post hoc test between groups 1 and 4. ^||^*p* < 0.05 one-way ANOVA, Tukey's post hoc test between groups 2 and 4. ^¶^*p* < 0.05 one-way ANOVA, Tukey's post hoc test between groups 3 and 4.

## Data Availability

All data generated/analyzed and used to support the findings of this study are included within the article.
